# A dementia education programme for pre-registration nurses: a protocol for process and outcomes evaluation

**DOI:** 10.1177/17449871241266805

**Published:** 2024-10-17

**Authors:** Deirdre Harkin, Aoife Conway, Assumpta Ryan

**Affiliations:** Lecturer in Nursing, School of Nursing and Paramedic Science, Faculty of Life & Health Sciences, Ulster University, Londonderry, Northern Ireland; Lecturer in Nursing, School of Nursing and Paramedic Science, Faculty of Life & Health Sciences, Ulster University, Londonderry, Northern Ireland; Professor of Ageing and Health, School of Nursing and Paramedic Science, Faculty of Life & Health Sciences, Ulster University, Londonderry, Northern Ireland

**Keywords:** curriculum design, dementia care, dementia education, nursing education, pre-registration nursing, workforce development

## Abstract

**Background:**

Dementia’s increasing prevalence, particularly among the ageing population, highlights the urgent need for specialised care. Nurses play a critical role, but often lack sufficient training, leading to low confidence in dementia care. The Dementia Education Programme (DEP) was launched to address this, offering a multi-level curriculum for student nurses.

**Aims:**

This paper presents a protocol for evaluating the DEP for nursing students. This study aims to assess the programme’s impact on students’ knowledge, attitudes and confidence while exploring the mechanisms influencing the programme’s implementation and effectiveness.

**Methods:**

The protocol employs a dual-fold evaluation, incorporating outcomes and process evaluation. The study uses a mixed-methods strategy, specifically a sequential explanatory design. Quantitative data are gathered longitudinally at four time points using validated instruments to assess knowledge, attitudes and confidence. Based on the results of the quantitative analysis, qualitative data is then collected through interviews and detailed field notes.

**Results:**

The DEP is described in detail, emphasising essential elements and underpinning theories. The protocol addresses ethical considerations as well as possible strengths and weaknesses of the study.

**Conclusions:**

By combining quantitative and qualitative methods, the research aims to provide a comprehensive evaluation, informing future educational practices and policy development in dementia care.

## Introduction

Dementia presents a significant global health challenge due to its rapidly increasing prevalence among individuals (The World Health Organization (WHO), 2023). Age is the primary risk factor for dementia, and as the ageing population continues to grow, the prevalence of this condition is anticipated to escalate exponentially in the forthcoming years (WHO, 2017). This gives emphasis to the urgent need for attention and action to address the impact of dementia on individuals, society and the healthcare system.

In the healthcare sector, there is an escalating demand for professionals who can provide specialised care and support to those living with dementia. Quality nursing care, in particular, is crucial for addressing the healthcare needs and enhancing the quality of life for people living with dementia (Bail and Grealish, 2016; Dookhy and Daly, 2021; Gibson et al., 2021). Nurses in various healthcare settings frequently encounter individuals living with dementia and others who are impacted by dementia (Dening, 2019; Harkin et al., 2022). The nursing role in dementia care is evolving with the need to manage more complex situations, including multimorbidity, intricate medication plans and cognitive and behavioural symptoms of dementia (Gibson et al., 2021; Pan et al., 2022). However, literature suggests that nurses may lack sufficient training in dementia care, resulting in low confidence levels and, at times, negative attitudes towards those living with dementia (De Witt and Ploeg, 2016; Evripidou et al., 2018). Martin et al. (2020) examined the gaps and priorities in dementia care across Europe and concluded that inadequate care provision is partly due to a lack of knowledge, skills and confidence in meeting the needs of individuals with dementia, as well as varying attitudes towards providing care for people with dementia. The World Health Organization has identified the essential need to transform and upscale education and training of the health and social care workforce ensuring that nurses have the knowledge, skills and competencies relevant to the needs of people with dementia and their carers (WHO, 2018). Higher Education Institutions (HEIs) bear a significant responsibility in equipping pre-registration nurses to address this pressing need and enhance the capacity of the future dementia care workforce, ultimately improving the quality of care provided (Jack-Waugh, 2023; Martin et al., 2020; WHO, 2018).

A recent review undertaken by the authors of this paper synthesised the literature on dementia education within HEIs and explored possible contextual factors, mechanisms and outcomes shaping the success of dementia education interventions for nursing students. This review identified the need for dementia education with diverse modalities, emphasising personal narratives and emotional connections, alongside interventions featuring high fidelity, active engagement, reflective opportunities, knowledgeable instructors and peer support. In addition, the review highlighted gaps in the literature including the need to explore the effectiveness and outcomes of a sequential approach where the curriculum is structured in a way that each year builds upon the knowledge and skills acquired in the previous year(s) ensuring consistency and logical progression throughout the curriculum. This review also unearthed a significant gap in understanding the impact of individual characteristics (e.g. age, gender, ethnicity and prior experience) on the effectiveness of dementia education for registration nursing students. This lack of targeted analysis means that we currently cannot determine the optimal ways to tailor dementia education to meet the varied needs and backgrounds of individual learners.

This current paper details a comprehensive protocol guided by the under-pinning theory of implementation science and the Medical Research Council (MRC) framework. Throughout this paper we explore the details of the Dementia Education Programme (DEP; the intervention) and present a dual-fold approach to the evaluation, which encompasses both process and outcome evaluation. We will provide insights into the DEP, relevance of the study, study design and recruitment, as well as the strategies we will employ to collect and analyse data.

### Intervention

The DEP was launched in 2020 by the lead author of this paper. It is a mandatory component of the curricula for adult and mental health nursing in Ulster University, Northern Ireland, with the aim of ensuring workforce ready nurses who have the skills, knowledge and confidence to provide high-quality dementia care. The DEP is a systematically structured, multi-level curriculum tailored to progressively deepen students’ understanding of dementia and is aligned to the Dementia Learning and Development Framework Northern Ireland (HSCB, 2016) and underpinned by the principles of the Person-Centred Nursing Framework (McCormack et al., 2021). In the first year, Level 1 serves as an introductory stage, focusing on awareness and understanding. Level 2, delivered in the second year, students delve into more detailed aspects of dementia care and theory. By the third year, students engage with Level 3, accumulating specialised knowledge in dementia care. This sequential approach aims to provide a holistic and comprehensive education on dementia, with each level thoughtfully building on the previous one to foster a deep and compassionate understanding of the subject. The DEP is delivered by the authors of this paper, each of whom has a background in nursing and extensive experience in dementia care. Additionally, they are esteemed educators with substantial expertise in learning and teaching methodologies. The DEP also integrates interprofessional collaborations with local a healthcare trust and voluntary organisations, enhancing the sharing of expertise and community-relevant narratives.

### Essential elements of the DEP

**Figure 1.**
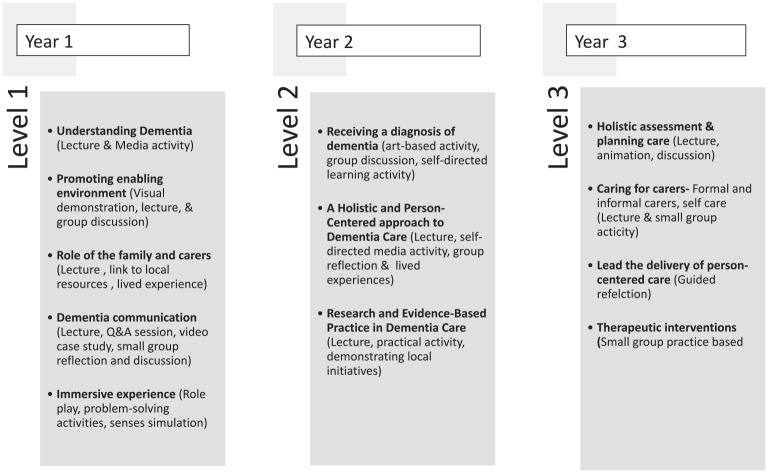
Essential elements.

Prior to commencement of this study a clear description of the DEP, its implementation plan, and expected outcomes was developed and shared with stakeholders. This was an iterative process that will continue throughout the study. Although some ambiguity may persist due to feedback, reflection and keeping up to date with emerging evidence, the authors of this paper are committed to ongoing collaborating with key stakeholders to resolve any uncertainties (Moore et al., 2015). Essential elements of the programme have been identified (Horner et al, 2006; Figure 1). These have been informed by reviewing the literature available on dementia education programmes, consultation with the underpinning framework (HSCB, 2016) and with expert colleagues (Haynes et al., 2016). Each component of the programme is conducted through face-to-face sessions on campus. In addition to these sessions, students are expected to engage in self-directed activities, which they can complete at their own convenience, either before or after the in-person meetings, in a time and location that suits them best.

**Figure 2.**
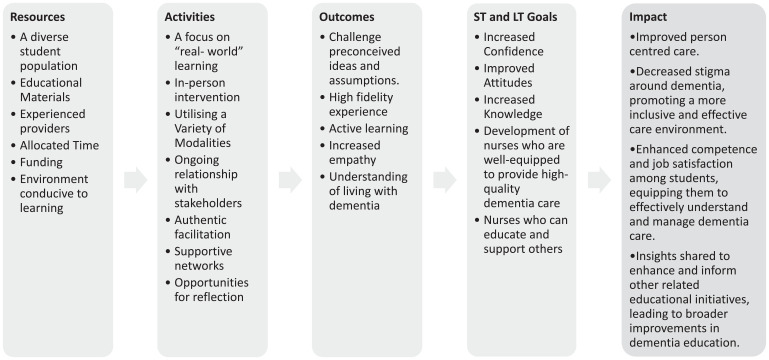
Logic model.

As seen in Figure 1, there are multiple interacting elements within the DEP. There is complexity not only within the intervention itself but also in the interactions between these elements (Campbell et al., 2000; McGill et al., 2020; Skivington et al., 2021). In addition, there is complexity within the context into which the DEP is introduced (Craig et al., 2008; McGill et al., 2020; Skivington et al., 2021). To identify core questions for this evaluation, we began by listing causal assumptions which was informed by a review of the literature, consultations with stakeholders and discussions within the research team. This process informed the logic model, as depicted in Figure 2.

## Aim

This study seeks to assess the influence (if any) of the DEP on the knowledge, attitudes and confidence of nursing students. Additionally, it aims to delve into the mechanisms that shape the implementation of the programme.

## Objectives

1. To investigate pre-registration nursing students’ knowledge, attitudes and confidence in dementia at four time points over a three-year degree using validated instruments.
2. Explore participants views and experiences of the DEP via semi-structured interviews and field notes.
3. Gain an insight into the processes undertaken, elucidating the underlying reasons and mechanisms that contributed to specific outcomes.

## Participants and recruitment

All students who meet the inclusion criteria will be invited to participate the study (must be a pre-registration nursing student at the participating university undertaking the BSc Hons/BSc Nursing Degree programme (Adult or Mental Health 2022 intake). Students will be provided with information via email on the study. Consent will be assumed on completion of the questionnaire; this is detailed on the information email. There are currently 430 students enrolled in these programmes. Sample size was calculated ensuring a margin of error of 5%, a confidence level of 95%, using the total population size of 430 and an anticipated response distribution of 50%. To fulfil the aims and objectives of this study, we aim to recruit 204 participants (minimum).

## Study design

This protocol paper explains the methods and rationale of two evaluation studies running simultaneously: outcomes evaluation and process evaluation. These two types of evaluation serve different, but complementary purposes in providing a comprehensive view of the programme whilst rigorously assessing the effectiveness of the DEP.

An outcomes evaluation has been chosen for its ability to determine whether the programme has achieved its intended goals and objectives. Although this is essential to understand, there is a strong focus within the literature to go beyond what works to explain discrepancies between expected and observed outcomes, to understand how context influences outcomes, and to provide insights to aid implementation (Craig et al., 2008). Process evaluation goes beyond considering ‘what works’ to understand how and why an intervention works (or does not work) in real-world settings. This information can be valuable for refining and optimising interventions to improve their effectiveness and impact. This study will follow the guidelines of a process evaluation outlined by Moore et al. (2015) to uncover the causal assumptions underpinning the intervention and to understand if the intervention works (or does not) to build an evidence base that informs policy and practice. It looks at how the programme is delivered and whether it is being implemented as intended. During the process evaluation, any adjustments, tweaks or changes made will be noted to ensure transparency and to understand how they may have impacted the study’s implementation and outcomes. This includes noting any changes in the delivery of the intervention or any other procedural elements. Including these adjustments in the evaluation helps in assessing the fidelity of the intervention and provides insights into potential areas for improvement.

Running these two types of evaluation simultaneously aims to provide a comprehensive view of the programme and will allow for a more holistic understanding of the programme’s success and can help identify areas for improvement.

### Understanding context

Context, as defined by Linnan and Steckler (2002), encompasses aspects of the larger social, political and economic environment that can affect intervention implementation. Understanding and assessing the context in which the DEP operates is essential to understanding how it works. To systematically account for context and avoid overlooking potentially significant dimensions, the study draws from published guidance by Craig et al. (2018) on considering context in population health intervention research. This guidance serves as a set of prompts to ask critical questions, which are essential to identify key features of the context that might impact the intervention’s success. Throughout this study, in order to capture the context, we will ask the following questions:

– What are the sociodemographic characteristics of the recruited participants and how might these factors influence the programme’s implementation and outcomes?
– Who are the key stakeholders and their roles within the intervention context? How do their interests and influence affect the programme’s success?
– Are there any economic or financial factors in the intervention context that may impact the availability of resources for the programme?
– How does the physical environment, such as the availability of facilities and infrastructure, influence the programme’s implementation?
– Are there ongoing or previous interventions that might interact with or complement the DEP, and how might these interactions influence outcomes?

### Understanding implementation

MRC guidance on the process evaluation of complex interventions suggests the need to investigate the reach, dose delivered, dose received, fidelity, implementation and recruitment (Linnan and Steckler, 2002). The questions below address these recommendations:

– *Reach*: What proportion of the intended target audience participated in the DEP?
– *Dose Delivered*: Were all the essential elements of the programme delivered to the participants as intended? Were there any deviations, and if so, what were the reasons for these deviations?
– *Dose Received*: To what extent did participants engage with and actively participate in the DEP components? Did participants demonstrate consistent participation throughout the programme, or were there variations in their engagement levels? Were there any observed barriers or facilitators to participants’ ability to receive and engage with the essential components?
– *Fidelity*: To what extent did implementers (lecturers/facilitators) adhere to the programme? How well were the intervention activities executed in terms of quality and competence?
– *Implementation*: Were there any best practices or successful approaches that emerged during implementation? Were there any obstacles or challenges that hindered the smooth implementation of the programme?

In this evaluation, our goal is to comprehensively assess the implementation of the DEP and the factors influencing its success or lack thereof. By combining qualitative and quantitative research methods, it is intended to explore the different aspects of the programme, how it functions in the real world and the implications of changes in its implementation (McGill et al., 2021; Moore et al., 2015).

## Methods

### Quantitative data

This study will apply longitudinal methods to explore change (relating to students’ knowledge, attitudes and confidence on dementia) over time (3-year degree). This study will compare participants’ knowledge, confidence and attitudes towards dementia before and after the intervention, to see what’s changed (Hopkins et al., 2020).

Using a repeated design, we will gather quantitative data at four different time points, prior to delivery of any training and post-delivery of each level. A data collection questionnaire has been devised compromising of three validated tools to measure knowledge (Dementia Knowledge Assessment Scale (DKAS; Annear et al., 2017)), attitudes (Dementia Attitudes Scale (DAS; O’Connor and Mc Fadden, 2010)) and confidence (Confidence in Dementia Scale (CODE; Elvish, 2014)). An in-depth review of existing literature took place to identify instruments that have been used successfully to measure similar constructs (e.g. Cheston et al., 2016; Parveen et al., 2017; Sullivan and Mullan et al., 2017), and consideration was given to the appropriateness of the target population (Aljezawi et al., 2022; Mastel-Smith et al., 2020; Mulyani et al., 2023). The selection of the final tools was narrowed down based on research objectives and the validity and reliability of each tool and shared within the research team and expert stakeholders for open discussion. In addition to the three tools participant demographics (such as age, gender, ethnicity and experience in dementia care) will be added and put into a single questionaire.

### Pilot testing

The combination of the approved, validated tools, coupled with demographic questions, underwent a pilot phase involving 21 nursing students in the second year of the degree programme at the participating university. The nursing students were invited to participate in the pilot and were subsequently asked to provide feedback on their experience with the survey. All feedback was documented and reviewed to identify successful aspects and pinpoint any necessary changes for improvement. The students confirmed that the questions were clear, easily understood and pertinent to the study. This process provided insights into the time typically taken to complete the survey, ensuring it strikes a balance between not being excessively time-consuming or rushed, as the students reported a completion time ranging from 7 to 10 minutes. This pilot phase served the purpose of validating the functionality of the online platform and confirmed that participants could navigate the survey without encountering any significant technical difficulties, which is crucial for the smooth execution of the study.

### Qualitative data

Semi-structured interviews will be conducted to explore participants’ views and experiences of the DEP. To gain an understanding into their perspectives, experiences, opinions and views consenting participants will be interviewed (Busetto et al., 2020). A purposeful sampling technique will be employed by inviting an expression of interest from all participants who have completed the DEP programme, ensuring inclusivity and reducing selection bias (Pannucci and Wilkins, 2010). Interviews will be held until no new significant information is revealed, ensuring that the data collected is comprehensive and reflects the diverse experiences and views of the population involved. This approach helps in capturing a rich, varied set of insights that accurately represent the different perspectives within the population group.

These interviews will be conducted by a member of the research team, who has been trained and has experience in qualitative interviews. A semi-structured interview guide will be devised by drawing from the study’s research questions and insights obtained from the quantitative data (Adeoye-Olatunde and Olenik, 2021). This triangulation of data from various sources aims to enhance the overall validity of the study and provides a more comprehensive insight into the broader educational context. Informed consent will be gained prior to each interview with the additional clause of giving consent to use a digital recorder for the interviews.

In addition, researchers will take field notes and keep reflective diaries as essential tools while actively participating in the DEP, taking on roles as educators or observers (Hamilton and Finley, 2020). Comprehensive field notes will encompass detailed and objective observations of the educational settings, capturing nuances in interactions, nonverbal cues and the overall atmosphere (Phillippi et al., 2018). These notes will serve as a valuable source of qualitative data, providing a snapshot of the programme’s dynamics. Concurrently, reflective diaries will offer a space for researchers to introspect, documenting personal thoughts, emotions and evolving perspectives. This dual approach aims to foster a deeper understanding of the researchers’ biases, assumptions and decision-making processes throughout the research journey (Mortari, 2015). A critical component of this methodology is the integration of reflexive analysis, involving the ongoing examination of the researchers’ roles, influences and potential biases (Olmos-Vega et al., 2023). By intertwining field notes, reflective diaries and reflexive analysis we aim to ensure that both the observed and the observer’s experiences are integral to the data collected.

### Data analysis

Following a sequential explanatory design, quantitative data will first be analysed using SPSS. Insights from this analysis will inform the development of the interview guide, aiming to explain or expand on the quantitative findings. Interview data will then be imported into NVivo11(Windows) for effective data management. Field notes and reflexive entries from the DEP programme will be organised chronologically to maintain the sequence of observations and can be referred to clarify, explain, interpret or raise questions about specific happenings. After transcribing interviews, researchers will use the field notes taken during the interview to ‘add back’ critical nonverbal content into the transcripts. Data from field notes, reflective diaries and other qualitative sources (e.g. interviews) will be triangulated to enhance the validity and reliability of the findings. This approach allows for a more nuanced understanding by cross-verifying information from multiple data points.

## Ethical considerations

This study has been approved by Research Ethics Committee application number: FCNUR-23-001.The primary ethical concerns pertain to the aspects of voluntary participation, obtaining informed consent and ensuring the confidentiality and protection of data. The email invitation and participant information sheets have been carefully written to address these ethical considerations. Participation in the study will be kept confidential. To ensure privacy, any personal data will be anonymised using a unique identification number, known only to the research team through an encrypted file. Participants’ identities will be anonymised in all publications and outputs, and all data will be securely held complying with UK GDPR standards. This is a low-risk study, and there is limited risk of distress to participants; however, a distress protocol has been devised. There may be a possibility that some individuals (whether through personal or professional experiences) may disclose experiences with issues pertaining to the Safeguarding Vulnerable Adults, in this case a disclosure protocol has been devised. All members of the research team have a professional background in nursing, along with significant experience in teaching and learning. The team members have expertise in conducting and overseeing research and have received research training. The involvement of research team members in the development and delivery of the Dementia Education Programme (DEP) may shape their perspectives and approaches, potentially introducing biases such as performance bias or confirmation bias. To address this, researchers will engage in reflexive practice, critically examining their biases and assumptions to ensure transparency and promote critical reflection within the team. This will uphold the integrity and validity of the study findings while adhering to ethical standards. Additionally, there is a risk of social desirability bias, where participant responses may be influenced by their connection to academic progression, given the dual role of the research team as educators and evaluators. To mitigate this bias, participants will be encouraged to respond truthfully, reassured that participation will not affect academic progression and reminded that all identifiable information will be anonymised.

## Discussion

This paper outlines the approach that will be taken to identify the potential impact of the DEP on nursing students’ knowledge, attitudes and confidence as well as explore the mechanisms that influence the successful implementation of the programme. The increasing numbers of people living with dementia, coupled with the current challenges confronted by healthcare providers in community and inpatient settings, where the demands of dementia care are continually rising, highlight the importance and pertinence of this research. Understanding the impact of educational interventions like the Dementia Education Programme is essential in addressing these challenges and providing high-quality, compassionate care for this population. Several studies have explored the outcomes of other dementia teaching and learning resources (e.g. Barros et al., 2020; Campbell et al., 2021; Daley et al., 2020; Grosvenor et al., 2021; Rossler and Tucker, 2022); however, this is the first study (to the authors’ knowledge) to evaluate a sequential approach in a way that each year builds upon previous year.

## Potential strengths and limitations

This study has several potential limitations that require consideration. Conducting the study in a single university limits the generalisability of findings to diverse educational contexts, as institution-specific characteristics may influence outcomes and implementation in different settings. Voluntary interviews also introduce the possibility of participant self-selection bias. Additionally, while the study provides valuable short-to-medium-term insights spanning 3 years, its capacity to assess the DEP’s long-term impact in practice is limited. However, this study also has many strengths, the application of implementation science principles and the established MRC framework aims to provide a rigorous evaluation of the DEP. This robust methodological foundation provides a systematic approach to assessing the programme’s effectiveness and implementation. The incorporation of mixed methods, combining quantitative measures and qualitative data, enriches the study’s comprehensiveness by allowing for a multifaceted understanding of the DEP’s impact. Rigour is further maintained through peer validation of qualitative data, ensuring that interpretations are accurate and credible. Ongoing reflexive analysis helps to identify and mitigate researcher biases, while rigorous scrutiny by the broader research team provides additional layers of oversight and critical evaluation. These elements collectively enhance the study’s validity and reliability, ensuring that the findings are both trustworthy and meaningful.

**Key points for policy, practice and/or research**

- There is an increased demand for healthcare professionals, especially nurses, who can provide care to people living with dementia. Quality nursing care is essential to improve the quality of life for people living with dementia.
- Despite the crucial role of nurses in dementia care, there is evidence that many nurses lack sufficient training and education, resulting in low confidence and, at times, negative attitudes towards people living with dementia. This inadequacy in education leads to gaps in care provision and affects the overall quality of dementia care.
- The Dementia Education Programme (DEP) is a mandatory, multi-level curriculum designed to progressively deepen nursing students’ understanding of dementia. This programme is structured to build on knowledge and skills year by year, aiming to produce workforce-ready nurses proficient in high-quality dementia care. It includes interprofessional collaborations and emphasises personal narratives and emotional connections.
- The proposed study employs both process and outcome evaluations to assess the DEP. This dual approach helps understand not only the impact of the programme on students’ knowledge, attitudes and confidence but also the mechanisms and context that influence its implementation and effectiveness.
- The comprehensive evaluation of the DEP has the potential to offer valuable insights into how structured, multi-level education can enhance nursing students’ knowledge, attitudes and confidence in dementia care. These findings can inform the development of more effective training programmes, guide future research and influence health and social care policy to better prepare nurses for the increasing demands of dementia care.

**Supplemental Material**

sj-pdf-1-jrn-10.1177_17449871241266805 – Supplemental material for A dementia education programme for pre-registration nurses: a protocol for process and outcomes evaluation Supplemental material, sj-pdf-1-jrn-10.1177_17449871241266805 for A dementia education programme for pre-registration nurses: a protocol for process and outcomes evaluation by Deirdre Harkin, Aoife Conway and Assumpta Ryan in Journal of Research in Nursing
